# Ultrasound-Assisted Extraction of Phenolic Compounds from *Adenaria floribunda* Stem: Economic Assessment

**DOI:** 10.3390/foods11182904

**Published:** 2022-09-19

**Authors:** Miguel Lopeda-Correa, Beatriz E. Valdés-Duque, J. Felipe Osorio-Tobón

**Affiliations:** Faculty of Health Sciences, University Institution Colegio Mayor de Antioquia (COLMAYOR), Carrera 78 #65-46, Medellín 050036, Colombia

**Keywords:** *Adenaria floribunda*, ultrasound-assisted extraction, phenolic compounds, economic assessment, cost of manufacturing

## Abstract

*Adenaria floribunda* is a native species found in tropical regions of South America used as a traditional medicine. Ultrasound-assisted extraction (UAE) is an extraction process known to increase the extraction yield, reduce extraction times, and use low temperatures. This study aims to obtain water-based extracts from A. floribunda stems using UAE, hot water extraction (HWE), and Soxhlet extraction and perform an economic analysis. The global extraction yield (GEY) and total phenolic compounds (TPC) of extracts ranged from 5.24% to 10.48% and from 1.9 ± 0.44 mg GAE g^−1^ DW to 6.38 ± 0.28 mg GAE g^−1^, respectively. Gallic acid, catechin, and ferulic acid were identified in the extract using HPLC-UV. Results indicate that Soxhlet extraction has the best performance regarding GEY and TPC. However, after performing an economic assessment, the cost of manufacturing (COM) of Soxhlet extraction (US$ 5.8 flask^−1^) was higher than the UAE (US$ 3.86 flask^−1^) and HWE (US$ 3.92 flask^−1^). The sensitivity results showed that obtaining extracts from A. floribunda by UAE and HWE is economically feasible when the selling price is above US$ 4 flask^−1^. Soxhlet extraction is a feasible technique when the selling price is above US$ 7 flask^−1^.

## 1. Introduction

The Global Biodiversity Information Facility reports *Adenaria* as one of the 28 genera belonging to the family Lythraceae, distributed geographically in the tropical regions of South America, Central America, and Western India. *A. floribunda* is a native species found in several countries in this region. One of these is Colombia, in which its presence has been reported in the states of Amazonas, Antioquia, and Chocó [1]. In Colombia, *A. floribunda* is known as clavelito, chaparral, guayabillo, coralito and chaparro [2]. Moreover, its main reported use is as wood for tool handles. Other applications include ornamental functions, wildlife food, and helping the recovery of degraded soil.

The lipidic composition of different genera in the Lythraceae family was described by Graham and Kleiman [3]. Linolenic acid is a dominant fatty acid in all genera assessed. In *Adenaria*, the second most abundant pattern is for lauric acid and oleic acid, which are present in almost equal amounts. A study on Peruvian medicinal plants involved extracts of *A. floribunda*, one of the eight species originating from the indigenous medicine (Shipibo-Conibo tribe) with traditional ethnomedical uses to treat diarrhea and rheumatism. In vitro and in vivo assays using a mouse model show the moderate anti-inflammatory activity of these plant extracts [4]. In a similar study with another tribe of the Peruvian Amazon (San Martin Quechuas), the medicinal use reported for *A. floribunda* involved it being crushed and mixed with warm water for treating constipation [5].

Several studies have focused on the potential of medicinal plants as sources of phytochemical and polypharmacology compounds present in different plant tissues such as leaves, seeds, flowers, stems, and roots [6,7,8]. A natural group of secondary metabolites of plants considered useful for its beneficial health effects in humans is phenolic compounds [9]. They also possess biological properties such as antioxidant, antibacterial, and anti-inflammatory effects [8,10]. However, some species to which the community has assigned a medical use are poorly investigated, and their phytochemical profile and biological activity of phenolic fractions remain unknown. In addition, *A. floribunda* is used to treat blood diseases and immune system disorders in some regions of Antioquia, Colombia [11]. For instance, people of some regions of Antioquia prepare *A. floribunda* stems in a way similar to making tea to treat the diseases mentioned above. In this context, water is chosen to extract phenolic compounds from *A. floribunda* stems. Water is considered one of the greenest solvents. Furthermore, water is non-toxic, and as the infrastructure for its transportation is already established, it has minimum environmental impacts [12].

Phenolic compounds can be used in the food and pharmaceutical industries to replace synthetic compounds. For instance, incorporating phenolic compounds can impact the overall characteristics of food and pharmaceutical products. Moreover, they can be used as an ingredient to develop functional foods due to their bioactive properties [13]. However, the recovery of phenolic compounds from vegetal sources is challenging because of their instability. Conventional extraction methods such as maceration, infusion, and Soxhlet have been used for the extraction of phenolic compounds. However, these extraction techniques use long extraction times which can trigger the degradation of the compounds, and the yields are lower. Moreover, toxic solvents or non-GRAS solvents are used in conventional extraction techniques [14]. Therefore, to overcome these drawbacks, it is crucial to use alternative extraction techniques that can obtain high extraction yields during the process under extraction conditions that avoid degradation of the compounds.

Ultrasound-assisted extraction (UAE) is an extraction technique that uses shorter extraction times, reduces organic solvent consumption and energy, and saves costs. Moreover, when UAE is compared with conventional extraction techniques, UAE is considered environmentally friendly [15]. In the UAE, cavitation is a critical phenomenon in the extraction process. Cavitation is an acoustic phenomenon where microscopic gas bubbles are formed in a liquid medium. These bubbles grow to reach an unstable size. The bubbles then reach an unstable radius and collapse, releasing a huge amount of energy (5000 K and 200 bar). The collapse accelerates the extraction process, enhancing the performance of the extraction processes [16]. For instance, when bubbles collapse near solid surfaces such as cell walls, releasing high pressure and temperature generates the breakdown of the cell walls. Then, the compounds are released into the extraction solvent [17].

However, there are no reports amongst the UAE studies of vegetal sources on the use of UAE in the extraction of *A. floribunda* stems. Moreover, to encourage the cultivation of species such as *A. floribunda* and the use of UAE to obtain extracts with bioactivity, it is crucial to perform an economic assessment and sensitive analysis of the extraction process. Therefore, this work will promote the exploitation of wood crops and their byproducts, and establish the viability of obtaining bioactive extracts from A. *floribunda* stems. Consequently, this study aimed to evaluate the acquisition of bioactive extracts from A. floribunda stems by the UAE, Soxhlet, and hot water extraction. Additionally, the extraction processes were evaluated in economic terms. The model used in the economic assessment included the evaluation of different selling prices and a comparison among the extraction processes.

## 2. Materials and Methods

### 2.1. Raw Material

The *A. floribunda* stems were collected near Urrao City (Antioquia, Colombia). The raw material was cut into tiny pieces, shade-dried at room temperature, and ground into dust using a wood sawdust machine. Then, the raw material was sieved through an 80-mesh screen and stored in hermetically aluminum sealed bags at −22 °C before use.

### 2.2. Extraction Procedures

#### 2.2.1. Ultrasound-Assisted Extraction

A 750-W ultrasonic homogenizer (Cole-Parmer, Vernon Hills, IL, USA) was used to perform the UAE experiments. A quantity of 20 g of raw material was mixed with 200 mL of water to keep a solvent-to-feed ratio (S/F) of 10 for all extractions. A water bath (VWR, model WB05 International, LLC, Radnor, PA, USA) was used to control the temperature. Extraction was conducted in the pulse mode with 2 s OFF and 2 s ON. The experiments were performed using temperatures of 30 °C, 45 °C, and 60 °C, 2 min and 10 min of extraction time; and ultrasound amplitude of 20%, 40%, and 60%. The extracts were filtered through a filter (125-mm Advantech^®^) and kept at −22 °C in a 100 mL amber flask for further analysis. To determine the global extraction yield (GEY), an oven (Memmert UN110, Schwabach, Germany) was used to evaporate the solvent from the remaining extract at 80 °C for 48 h, according to the methodology described by Galviz-Quezada et al. [18].

#### 2.2.2. Soxhlet Extraction and Hot Water Extraction (HWE)

Soxhlet extraction was performed according to the literature [18]. A Soxhlet apparatus with a capacity of 500 mL loaded with 20 g of milled raw material and 400 mL of water was used to perform the Soxhlet extraction. The extraction was conducted at atmospheric pressure of 95 °C for 6 h. Hot water extraction (HWE) experiments were performed using 20 g of A. floribunda in a beaker containing 200 mL of boiling water using a heating magnetic stirrer (VELP Scientifica, Shanghai, China) at 130 rpm for 2 min and 10 min. The extracts were filtered through a filter (125-mm Advantech^®^) and kept at −22 °C in a 100 mL amber flask for further analysis. Soxhlet and HWE extractions were performed in duplicate. The solvent was evaporated using the same conditions previously mentioned to determine GEY.

### 2.3. Determination of Total Phenolic Content (TPC)

The Folin–Ciocalteu reagent method reported by Pacifico et al. [19] with some modifications was used to determine the total phenolic content (TPC) in the extracts. First, in a falcon tube, 1 mL of 10% (*v/v*) Folin-Ciocalteu reagent (Merck, Darmstadt, Germany) and 1 mL of the crude filtered extract diluted to 1:20 (*v/v*) were mixed. Next, the mixture was shaken and incubated in the dark at 30 °C for 5 min. After that, 5 mL of Na_2_CO_3_ (5% *w/v*) were added and the mixture was incubated for 90 min in the dark at 30 °C. A spectrophotometer (Thermo Fisher Scientific, Madison, WI, USA) at 760 nm was used to read the absorbance. A calibration curve with concentrations ranging between 0 and 20 mg L^−1^ was used. TPC was expressed in mg gallic acid equivalents (GAE) per g of dry weight (mg GAE g^−1^ DW). The analysis was performed in duplicate.

### 2.4. High-Performance Liquid Chromatography (HPLC) Analysis

Identification of PC in the extracts was performed by HPLC instrumentation (UltiMate 3000, Thermo Scientific™ Dionex™, Bremen, Germany). Before HPLC analysis, the samples were filtered using a nylon syringe filter of 0.45-μm (Sartorius, Göttingen, Germany). A ReproSil-Pur 120 C−18 column (5 μm, 250 × 4.6 mm) and a variable wavelength detector system (280 and 320 nm) were used to analyze the samples by HPLC. A gradient mixture (mobile phase) of water with 0.1% formic acid (solvent A) and acetonitrile (solvent B) was used. The gradient program of the samples was as follows: 0–10 min, 2–20% of B, 10–15 min, 20–30% of B, 15–20 min, 30–35% of B, 20–30 min, 35–30% of B, 30–35 min, 30–2% of B. The samples were analyzed using a flow rate of 1.3 mL min^−1^ at 35 °C. The injection volume was 20 µL. To identify the bioactive compounds in the extract, catechin, ferulic acid, syringic acid, coumarin, gallic acid, caffeic acid, chlorogenic acid, and quercetin were used as standards (Sigma–Aldrich, St. Louis, MO, USA).

### 2.5. Process Simulation Model

**Figure 1:**
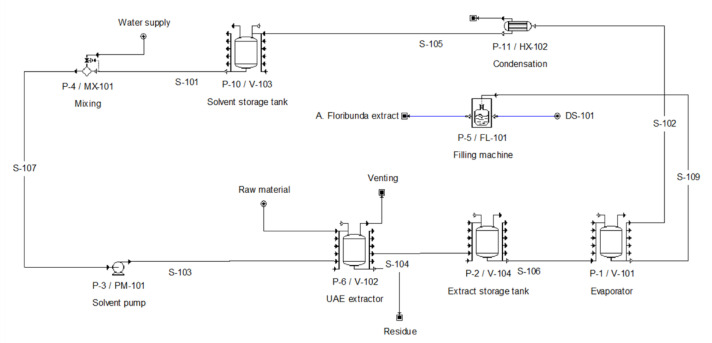
Process diagram of the UAE process.

SuperPro Designer 8.5^®^ (Intelligen Inc., Scotch Plains, NJ, USA) was used to conduct the UAE simulation. The flowsheet of the batch extraction processes is shown in Figure 1. The simulation process included a solvent storage tank (P-10/V-103), a solvent replacement inlet (P-4/MX-101), a pump (P-3/PM-101) to transport the solvent into an extractor (P-6/V-102), an extract storage tank (P-2/V-104), an evaporator (P-1/V-101), a condenser (P11/HX-102) and a flask filling machine (P-5/FL-101). The conditions for the condenser and evaporator were established according to the works published by Vieira et al. [20] and Veggi et al. [21].

### 2.6. Economic Evaluation

The value of the extraction units was provided by Shanghai Better Industry Co., Ltd. (Shanghai, China). The extraction and concentration units were considered with one extraction vessel with a volume of 300 L. The extraction unit could perform the three extraction processes evaluated in this work. When performing HWE and Soxhlet, the ultrasonic probe was OFF, and only the temperature and extraction time were set in the equipment. Thus, the cost of the extraction unit is the same for all extraction processes. In this work, we took into account the fact that the extract is packed in flasks containing 100 mL of extract. Thus, COM was expressed in terms of US$ flask^−1^ instead of US$ kg^−1^. The COM was determined as the sum of three main components: direct, fixed, and general expenses. Moreover, the following five major costs: cost of raw material (CRM), fixed capital of investment (FCI), cost of utilities (CUT), cost of waste treatment (CWT), and cost of operational labor (COL) were used to determine the COM.

FCI is related to the expenses involved in implementing the extraction unit and auxiliary equipment. CRM includes the costs of the preprocessing steps needed to prepare the raw material for extraction and the extraction solvent (water) costs. The CRM (*A. floribunda* sawdust) was presumed to be zero because it is a byproduct of the tool handle industry. The wage and number of operators of the UAE process are related to the COL. In this work, calculations were based on two operators operating the 300 L equipment. The CUT takes into account the energy used in the solvent cycle for heating and refrigerating and the use of electricity. The CWT was considered to be zero in this work because the residue generated from the extraction process is harmless and clean. For instance, the extraction residue can be disposed of as vegetable waste or used in other extraction processes. Direct costs (e.g., buildings, electrical facilities, instrumentation, insulation, and installation) and indirect costs (e.g., engineering, administrative rates, insurance, construction, cleaning services, and human resources) are estimated by the software. Thus, both costs were considered in the economic assessment.

This work estimated that the yield and extract composition obtained on a laboratory scale could be obtained on a 300 L scale using the same processing conditions (e.g., solvent, time, temperature, amplitude, and S/F ratio). The processes were designed to operate for 7920 h year^−1^ in three daily shifts for 330 d year^−1^. The amount of raw material processed was calculated based on the extractor vessel volume and the S/F ratio. The loss of solvent during the process was assumed to be 2%, which was the loss from the evaporator used by the simulator. Table 1 shows the data used to estimate the COM.

### 2.7. Experimental Design and Statistical Analyses

A complete randomized factorial design (2 × 3 × 3) with two replicates was conducted to evaluate the effect of process parameters in the UAE process. An analysis of variance (ANOVA) at a significance level of 0.05 was performed using the software Minitab^®^. In addition, a Tukey’s test was performed to evaluate significant differences among the different extraction processes.

## 3. Results and Discussion

### 3.1. Effect of the Extraction Process on GEY

**Figure 2:**
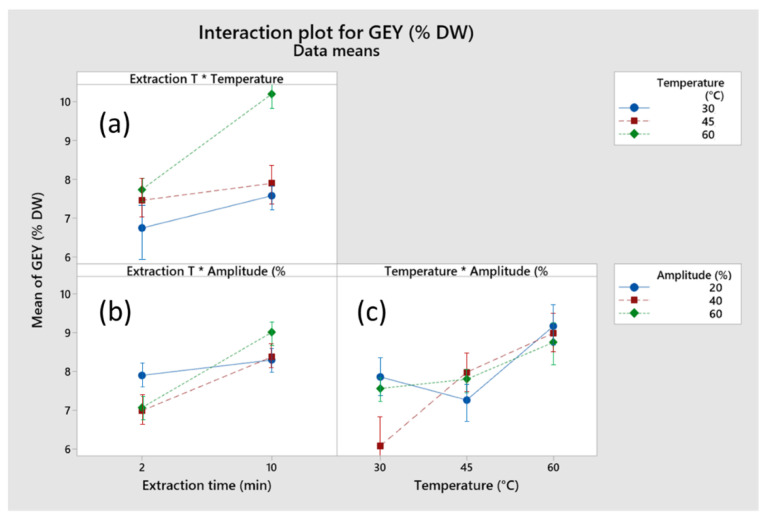
The effect of process parameters on GEY. (**a**) temperature and time, (**b**) amplitude and time, and (**c**) temperature and amplitude.

The effects of process parameters (amplitude, extraction time, and temperature) on GEY were evaluated in this work. Table 2 summarizes the results for GEY and total phenolic content (TPC). The maximum GEY (0.48 ± 0.34%) was obtained at 60 °C, 10 min and 60% amplitude, whereas the minimum GEY (5.24 ± 0.34%) was obtained at 30 °C, 2 min, and 40% amplitude. These results are similar to the solid-liquid extraction of phenolic compounds from lignocellulosic materials. For instance, Xavier et al. [25] obtained GEY between 5.38% and 10.45% in the solid-liquid extraction of pine and eucalyptus forestry byproducts using aqueous ethanol solutions. In another work, Fernández-Agulló et al. [26] studied bioactive compounds obtained from walnut biomass residues by solid-liquid extraction using water and ethanol. In their work, GEY ranged between 3.43% and 7.04%. As shown in Figure 2, when the extraction parameters increased, the GEYs also increased. The interaction between the extraction parameters was significantly tested at the significance level of *p* = 0.05 over the extraction time, temperature, and amplitude range studied. For instance, the interaction between extraction time × temperature (*p* = 0.004), extraction time × amplitude (*p* = 0.034) and, temperature × amplitude (*p* = 0.014) significantly affected the GEY. The results obtained in this work are similar to other studies in the literature. For instance, Cassiana et al. [27] used UAE to extract clove leaves (*Syzygium aromaticum*) with ethanol as the solvent and obtained extraction yields ranging from 5.79% to 11.95%.

Figure 2 shows the interaction plot of process parameters on the GEY. As shown in Figure 2, a higher GEY was obtained when the process was performed at 60 °C. As shown in Figure 2a, the combination of the higher temperature and longer extraction time caused an increase in the GEY. This behavior is due to increased mass transfer rates caused by temperature and extraction time. According to Pingret [28], in the UAE process, the combination of higher temperatures and longer extraction times may increase the GEY. In this case, with more cavitation bubbles and larger solid–solvent contacts, the solvent diffusivity is enhanced, and more compounds are recovered from the raw material. Similar behavior was observed in the interaction of extraction time and amplitude (Figure 2b). For both extraction times evaluated, the extraction yield of GEY increased with increasing amplitude. At higher amplitudes, the GEY substantially increased. For instance, the violent bubble collapse process is caused by an increment in amplitude. Thus, the sonochemical effect causes an increase in extraction efficiency [17]. On the other hand, the interaction between temperature and amplitude is complex. As shown in Figure 2c, the GEY increased due to the increase in temperature when the extraction process was conducted at an amplitude of 40%. Moreover, a slightly higher GEY was obtained when the extraction process was performed at an amplitude of 60%. In contrast, the GEY decreased at lower amplitudes when the temperature increased from 30 °C to 45 °C. However, the GEY performed better when the temperature continued rising to 60 °C.

Finally, a Tukey’s test was conducted to compare the UAE with Soxhlet and HWE processes. The results showed that when the UAE was processed at 60 °C and 10 min and the amplitudes of 20%, 40%, and 60%, the GEYs were similar to those obtained using Soxhlet (Table 2). On the other hand, the HWE is an extraction method with the lowest GEYs, especially at an extraction time of 2 min. Although HWE was performed at a high temperature (boiling water), the extraction time was not enough to allow the extraction of the compounds. This behavior was observed when the UAE was conducted using a shorter extraction time (2 min). Moreover, at an extraction time of 10 min in the UAE process, it is possible to reduce the extraction time required to obtain similar yields to Soxhlet. For instance, the extraction time of the UAE is 36 times shorter than Soxhlet extraction. Therefore, the UAE allows higher extraction yields in a shorter time. Consequently, the UAE is more efficient than Soxhlet extraction or HWE. This behavior has been observed in extracting bioactive compounds from *Lagenaria siceraria* using ethyl acetate as an extraction solvent [29]. In this work, the Soxhlet extraction showed less efficiency than the UAE. For instance, when the UAE was performed at 50 °C, 150 W, and 30 min, the GEY was 5.08%, but when Soxhlet extraction was performed for 60 min, the GEY was 3.2%.

### 3.2. Effect of Extraction Process on Total Phenolic Content

The total phenolic content (TPC) of the UAE, Soxhlet, and HWE is shown in Table 2. TPC was obtained between 1.90 ± 0.54 and 6.38 ± 0.28 mg GAE g^−1^ DW. These TPC values obtained from different extraction techniques are consistent with those from other studies that used lignocellulosic materials. For instance, Klaric et al. (2016) studied the extraction of bioactive compounds from milled European black alder wood [30]. They obtained a TPC of 7.31 mg GAE g^−1^ DW. However, that result was obtained using water as an extraction solvent after cold maceration for 6 h at 20 °C. In another study, salt-based aqueous two phase systems were used to recover phenolic compounds from *Eucalyptus globulus* wood waste [31]. They obtained a TPC of 18.9 mg GAE g^−1^ DW after 90 min of extraction at 65 °C using a polyethylene glycol (PEG) 2000/phosphate salt-based aqueous two-phase systems. Thus, as in several plant materials, the TPC varies according to different factors such as plant species, tissue, and stage of maturity. Moreover, these TPC values from various extraction techniques are slightly lower than those from other studies that obtained phenolic compounds from other vegetal sources. For instance, a TPC of 7.14 mg GAE g^−1^ DW was obtained to extract phenolic compounds from *Stevia rebaudiana* leaves by pressurized liquid extraction using water as an extraction solvent [32]. Regarding UAE, Guandalini et al. [33] studied the extraction of phenolic compounds from mango peel. They obtained TPCs between 6.0 and 8.6 when the extraction was performed in an ethanol/water mixture at 30 °C for 8 min at 75% ultrasound intensity. The results obtained were lower than [34], who obtained phenolic compounds from *Retama sphaerocarpa* stems. However, in that study, the raw material was extracted with dichloromethane for 6 h by Soxhlet extraction. Extraction was then performed in a methanol/water mixture of 50/50 (*v/v*) at room temperature for 24 h with constant stirring.

**Figure 3:**
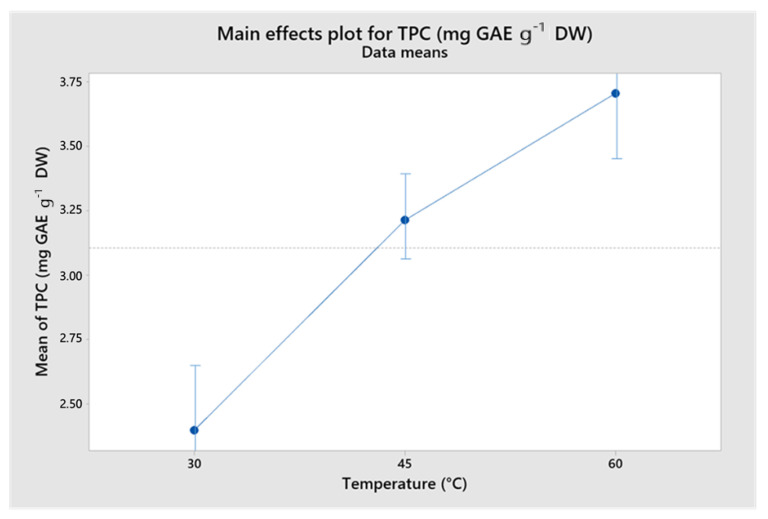
Main effects of the process parameters on TPC.

Among the different process conditions evaluated by the UAE, the temperature was the main factor affecting the extraction of phenolic compound. After a statistical test at the significance level of *p* = 0.05, no interactions were observed among the factors when considering the effects of the process parameters on the TPC. Moreover, only the effects caused by temperature were significant (*p* = 0.000). Thus, the effects of temperature on TPC were analyzed. As shown in Figure 3, the TPC also increases with increasing temperature. This behavior can be explained by the fact that the temperature increases the mass transfer rates of the phenolic compounds from the plant matrix into the solvent. This behavior was also observed by Xavier et al. [35] in the extraction of phenolic compounds from *Eucalyptus* wood wastes. After evaluating temperatures between 25 °C and 65 °C, the highest TPC was obtained at the highest temperature, 65 °C.

On the other hand, after performing a Tukey’s test, the highest TPC was obtained with Soxhlet extraction. Generally, phenolic compounds are degraded at higher temperatures, such as those used in Soxhlet. However, in this work, the behavior was different due to the characteristics of the matrix. For instance, as *A. Floribunda* stems are rich in lignocellulosic polymers, it is possible to break down the cells and release phenolic compounds only after a long extraction time at high temperatures. Compared to UAE, the combination of process parameters was inappropriate, rupture of plant cells was incomplete, and sufficient contact between the solvent and the matrix cell was not achieved in order to reach total solvent penetration for solubilization of the cell compounds. According to Tukey’s test, the TPC of Soxhlet extraction was significantly higher than that of UAE and HWE. For instance, TPCs obtained by Soxhlet extraction are about 35% higher than those obtained from UAE and HWE. However, although Soxhlet extraction performed better than UAE and HWE, these extraction techniques used only 2.7% of the extraction time required by Soxhlet. This suggests that further research is needed to increase the recovery of phenolic compounds from lignocellulosic materials.

### 3.3. HPLC Compound Identification

**Figure 4:**
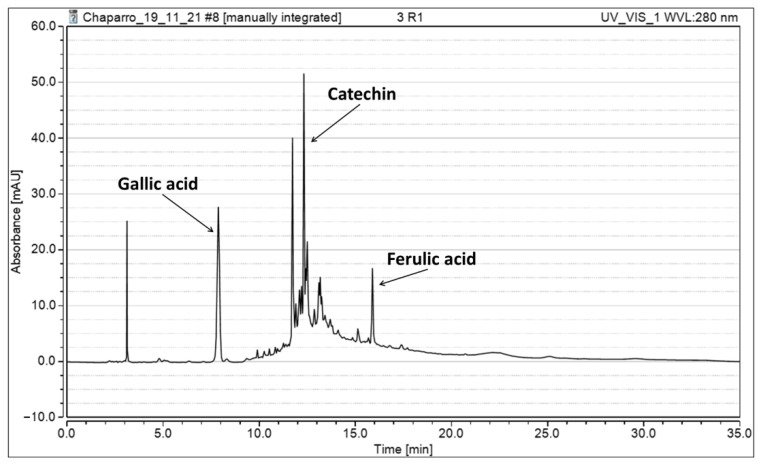
Chromatogram corresponding to the extract obtained by UAE at 60 °C for 10 min and 60% amplitude.

An HPLC analysis was carried out to identify the chemical components of the *A. Floribunda* extract. As shown in Figure 4, gallic acid, catechin, and ferulic acid were identified in the extract obtained, using UAE at 60 °C for 10 min and 60% amplitude. The phenolic compounds identified in the extract are recognized by their bioactivity. For instance, gallic acid is one of the major phenolic acids found in several plants and foods. Gallic acid is known for its antibacterial, antiviral, anti-inflammatory, antimutagenic, antioxidant, and anticancer properties [36]. Gallic acid can be used in the skin and leather industry as a chelating agent and the synthesis of trimethoprim, an antimicrobial agent used as a preservative in food and beverages [37]. A glucoside of gallic acid (glucogallic acid) was identified in extracts from leaves and stems of Red-osier Dogwood using hydrothermal extraction [38]. This glycosylation enhances the solubility of phenolic compounds in water. Catechin has a notable antioxidant activity, and it can act against diseases such as cancer and cardiovascular and neurodegenerative diseases. Moreover, catechins have anti-inflammatory and antioxidant properties. Catechins also enhance the performance of skeletal muscle [39]. The presence of catechin has already been identified in other lignocellulosic materials. For instance, Vázquez et al. (2012) reported catechin in eucalyptus bark extracts [40]. Finally, ferulic acid is one of the most abundant phenolic acids in plants. Ferulic acid has excellent action in the cell wall, protecting against UV radiation [41]. Ferulic acid has anti-inflammatory, antimicrobial, anticancer, anti-arrhythmic, and antithrombotic properties. This phenolic compound is widely used to delay skin photoaging processes and is a photoprotective agent in skincare formulation [42]. Ferulic acid has been reported by Guetchueng et al. (2020) in extracts from the stem bark of *Pseudospondias macrocarpa* [43]. In their study, Soxhlet extraction was used with n-hexane, dichloromethane, and methanol. Thus, these phenolic compounds can be found in other lignocellulosic materials, and their presence indicates that *A. Floribunda* extracts have potential applications in the food and pharmaceutical industries.

### 3.4. Economic Assessment

The COM of the extracts was determined using SuperPro Designer 8.5^®^ as a reference flask instead of kg. For the UAE, the COM was calculated under the following process conditions: 60 °C for 10 min and 60% amplitude. For HWE and Soxhlet, the processes were simulated at 95 °C for 10 min for HWE and 360 min for Soxhlet. For the UAE, HWE, and Soxhlet, the COMs obtained were US$ 3.86 flask^−1^, US$ 3.92 flask^−1^, and US$ 5.8 flask^−1^, respectively. The COMs obtained for all extraction processes are competitive compared to some natural plant extracts available on the market. For instance, plant extracts of fig [44], ashwagandha root [45], and cramp bark [46] have a 100 mL flasks selling price per flask ranging between US 22 flask^−1^ and US$ 35 flask^−1^. Thus, the extracts obtained can have marketing potential because their COMs are up to 9.1 times cheaper than the plant extracts on the market.

On the other hand, the COM of the Soxhlet process was higher than the COM of the UAE and HWE. In the present study, the COM of Soxhlet extraction was 54.9% and 51.7% higher than those of UAE and HWE, respectively. Moreover, Soxhlet extraction takes 36 times longer than the UAE and HWE extraction processes. Thus, the longer the extraction time, the lower the productivity of the process. For instance, UAE and HWE produce almost 50% more flasks than Soxhlet per year. Consequently, the COM of Soxhlet is 48% higher than the COM of UAE and HWE. This behavior has been observed in other, similar studies. For instance, Ochoa et al. [47] studied the extraction of anthocyanins from purple yam by the UAE. In their research, the COM obtained for Soxhlet extraction was 212% higher than those obtained for the UAE process. On the other hand, generally, novel extraction processes have a lower COM than conventional extraction processes. However, in this study, the COM obtained by UAE and HWE was similar. For instance, the COM of the UAE was only 1.5% lower than that of the HWE. Thus, in the production of *A. floribunda* extracts, the use of UAE is not advantageous compared to the conventional extraction process HWE.

**Figure 5:**
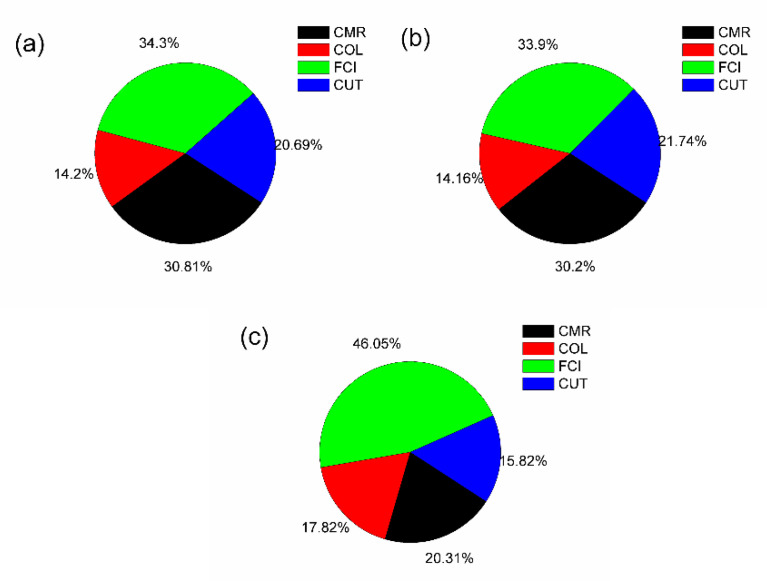
Contribution of COM components (CRM, CUT, COL, and FCI) to the A. floribunda extracts obtained by (**a**) UAE, (**b**) HWE, and (**c**) Soxhlet.

The major cost factors (CRM, COL, FCI, and CUT) on COM were analyzed for each extraction process (Figure 5). The FCI and CMR were the components with the greatest contribution to the COM for all extraction processes. For instance, the contribution of CMR to COM was 30.81%, 30.20%, and 20.31% for UAE, HWE, and Soxhlet, respectively. This result is similar to other studies with novel extraction processes. For instance, Aguiar et al. [48] studied the extraction of capsaicinoids from malagueta pepper using supercritical carbon dioxide (SFE). In their research, the CRM represents 63% of the COM, the cost of fresh pepper represents 94.85% of the CRM, and the cost of the extraction solvent (supercritical CO_2_) corresponds to 5.15%. Although we considered the cost of *A. Floribunda* sawdust to be zero, the preprocessing of the raw material represents around 60% of CRM. In contrast, the cost of the extraction solvent (water) and flasks represent 1% and 39% of the COM, respectively. Thus, since the preprocessing cost is a difficult parameter to reduce in the extraction processes evaluated in this study, we believe that COM can be reduced by using inexpensive flasks.

As can be observed in Figure 5, after CRM, FCI was a component with outstanding contributions to the COM. Among all extraction processes evaluated, the contribution of FCI to the COM was higher in Soxhlet extraction. As mentioned before, Soxhlet has the longest extraction time, the number of batches performed is lower, and when compared to UAE and HWE, less equipment is used. Therefore, FCI represents a larger percentage of COM composition in Soxhlet extraction. On the other hand, the contribution of FCI to the COM of UAE and HWE is similar. As the extraction time and yield are similar, the number of batches and productivity are also similar. As for the CUT, the contribution of the cost of this component to the HWE is slightly higher than the UAE. Although both extraction processes have similar extraction times and yields, more energy is required as HWE is performed in boiling water (95 °C). Consequently, the contribution of CUT to the COM of HWE increases. On the other hand, the contribution of CUT to the COM of Soxhlet was 40% lower than that in the UAE and HWE. This result seems inconsistent because Soxhlet extraction is a high-energy extraction process, and the CUT contribution should be higher. However, as mentioned before, the longest extraction time distracts the contribution of the costs of major components. Thus, this factor can affect the contribution of the costs of major components, depending on extraction time in the equipment that can perform different extraction processes.

The study of the COM components revealed that FCI is the main component in the final manufacturing costs, followed by CRM, labor, and utilities. For instance, at a lower COM, FCI represents about 90% of total COM. Consequently, to reduce COM, it is necessary to consider low-priced materials for equipment and plant implementation manufacture. Finally, the contribution of COL to the COM represents around 15% of the total COM for all extraction processes. As we considered the same number of workers per process, all extraction processes were designed to operate in three daily shifts for 330 d year^−1^. Therefore, for all extraction processes, the workers have the same period of employment, and thus, the wage value is the same.

### 3.5. Sensitivity Study

To determine a minimum selling price of the A. Floribunda extract for all extraction processes, a sensitivity study was performed. The selling price of the extracts was chosen around the COM obtained for all extraction processes. Therefore, we tested selling prices ranging from US$ 4 flask^−1^ to US$ 10 flask^−1^. Although almost all project indices were positive, simulation of the extraction process at the lowest selling price showed that none of the extraction processes were feasible in the UAE and HWE (Table 3). Of the three extraction processes, however, Soxhlet showed the worst-case scenario. For instance, gross margin is the percentage of the amount of sales per flask that a company can retain as gross profit [49]. In this case, when the selling price of the *A. floribunda* extract was US$ 4 kg^−1^, the gross margin was −45.77%. Therefore, the company will lose US$ 0.46 for the income generated. In the same way, project indices such as internal rate of return and net present value were not calculated, or they were negative. However, when the selling price of the product increases, the gross margin of all extraction processes also increases. For instance, when the selling price increased from US$ 4 kg^−1^ to US$ 10 kg^−1^, the gross margin increased from 3.43 to 61.37, 1.95 to 60.78, and −45.77 to 41.69 for UAE, HWE, and Soxhlet, respectively. In this study, the extraction process with a higher gross margin was UAE, followed by HWE and Soxhlet. Therefore, the gross margin of UAE and HWE is almost 50% higher than Soxhlet at higher selling prices. For instance, for UAE and HWE, the company will retain up to US$ 0.60 for every 1$ generated, and for Soxhlet extraction, the company will retain US$ 0.42 for every 1 US$ generated.

The annual profit generated by a unit of invested capital is known as the return of investment (ROI). Therefore, the higher this parameter, the higher the profitability of the project [50]. However, ROIs ranging between 10% and 15% are acceptable. In this study, when evaluating the lowest selling price, the profitability of all extraction processes was not acceptable. However, when the selling price was higher than US$ 4 flask^−1^, the ROI started to be acceptable. For Soxhlet extraction, the process only started to be feasible according to ROI, when selling prices were above US$ 7 flask^−1^. Therefore, considering the ROI of all extraction processes, Soxhlet is still a less feasible extraction process.

The payback time is the period required for recovering the cost of an investment. Thus, the shorter the payback time, the faster the recovery of the initial investment. Payback times between seven and ten years are acceptable for investment projects. As shown in Table 3, UAE and HWE possess shorter payback times, whereas the Soxhlet extraction payback times are longer for all selling prices evaluated. For instance, in both UAE and HWE, when the selling price is above US$ 4 flask^−1^, the payback time begins to be acceptable. For Soxhlet extraction, when the selling price is above US$ 6 flask^−1^, the payback times become acceptable. Moreover, although the feasibility of all extraction processes is acceptable at higher selling prices (US$ 10 flask^−1^), the initial investment is recovered 2.2 times faster in both UAE and HWE. Thus, both UAE and HWE are more economical and attractive than Soxhlet.

Another economic parameter that allows the profitability of a project to be measured is the internal rate of return (IRR). There is no minimal IRR value to accept or reject a project; however, the higher the value of the IRR, the more feasible the project. According to IRR, the profitability of UAE and HWE is better than Soxhlet. For instance, when all extraction processes are simulated using a selling price of US$ 10 flask^−1^, the IRR for UAE and HWE is 80.55% and 79.55%, respectively, whereas it is 34.1% for Soxhlet. In other words, with a total capital investment of US$ 1,645,000, the UAE and HWE would earn up to twice as much, compared to a compound annual growth rate of only 34.1% for Soxhlet extraction. The value of future income compared to the initial investment is described by the net present value (NPV) [51]. This parameter allows the feasibility of a project to be established. The project is promising if the NPV is positive. On the other hand, the project must be rejected if the NPV is negative. In this research, all extraction processes had negative NPV values at selling prices of US$ 4 flask^−1^. However, for both UAE and HWE, the selling prices above US$ 4 flask^−1^ produced positive NPV values. Moreover, the higher the selling price, the higher the NPV value. For instance, in the UAE process, when the selling price increased from US$ 5 flask^−1^ to US$ 10 flask^−1^, the NPV increased from US$ 0.80 × 10^6^ to US$ 5.77 × 10^6^. Thus, to generate more earnings and improve the feasibility of all extraction processes, higher selling prices must be considered.

In brief, after the economic assessment of the production of A. floribunda with UAE, HWE, and Soxhlet, when selling prices above US$ 5 flask^−1^ for UAE and HWE and US$ 7 flask^−1^ for Soxhlet were used, the extraction processes became economically feasible. As the yield and extraction times of UAE and HWE were similar, the economic performance of both processes was also identical. Although the selling cost of the Soxhlet extraction product is 40% higher than UAE and HWE, this selling price can still be feasible because it is in the range of similar extracts sold on the market.

## 4. Conclusions

This study showed that conventional extraction processes such as HWE and Soxhlet and the novel UAE process perform similarly in obtaining extracts from *A. floribunda*. Process parameters such as temperature, extraction time, and ultrasound amplitude affect the GEY and TPC in the UAE process. The GEYs are similar to those obtained using Soxhlet when the UAE process was conducted at 60 °C for 10 min at any amplitude, whereas HWE achieved the lowest GEY. Although Soxhlet extraction performs better regarding the recovery of phenolic compounds, it is 36 times more time-consuming than the other extraction techniques and requires more energy. Of all extraction processes, the Soxhlet extraction process produces a higher COM and less economic feasibility. The COMs of the UAE, HWE, and Soxhlet are US$ 3.86 flask^−1^, US$ 3.92 flask^−1^, and US$ 5.8 flask^−1^, respectively. A sensitivity study showed that when simulating the extraction processes at higher selling prices (US$ 10 flask^−1^), Soxhlet’s economic parameters such as gross margin, return on investment, and IRR are up to 2.3 times lower than those of the UAE and HWE. On the other hand, the initial investment can be recovered 2.2 times faster in the UAE and HWE. However, according to the sensitivity study, obtaining extracts from *A. floribunda* by UAE, HWE, and Soxhlet is feasible at selling prices higher than US$ 7 flask^−1^.

## Figures and Tables

**Table 1:** Input economic parameters used in the process simulation to estimate the COM.

Fixed Capital Investment (FCI)	
UAE extraction plant ^a^	US$ 136,800
Filling machine ^b^	US$ 15,000
Depreciation rate ^c^	US$ 10%/year
Annual maintenance rate ^c^	6%/year
**Cost of operational labor (COL)**	
Wage ^d^	US$ 2.2
Number of workers per shift	2
**Cost of raw material (CRM)**	
Pre-processing *A. floribunda* stems	US$ 2/kg
Water ^e^	US$ 1.06/t
Amber glass flask bottle (100 mL) ^b^	US$ 0.5/flask
**Cost of utilities (CUT)**	
Electricity ^e^	US$ 0.19/kWh
Water steam (high pressure) ^f^	US$ 20/t
Water ^f^	US$ 1.06/t
CaCl2 solution (refrigerant fluid) ^f^	US$ 0.75/t
**Project financing**	
Debt funding ^f^	50%
Loan period ^f^	10 years
Loan interest ^f^	9%
Depreciation period ^f^	10 years
**Project financing**	
Income taxes ^f^	40%
Product failure rate	1%

^a^ Shanghai Better Industry Co., Ltd. ^b^ Direct quotation ^c^ Based on Peters et al. [22]. ^d^ Based on Wage Indicator Foundation [23]. ^e^ Based on Empresas Públicas de Medellín, [24]. ^f^ SuperPro Designer 9.0^®^ Database.

**Table 2:** Global extraction yields (GEY) and total phenolic content (TPC) obtained from A. floribunda stems under UAE, HWE, and Soxhlet. Extractions were performed in duplicate.

Time	Temperature	Amplitude	GEY	TPC
(min)	(°C)	(%)	(% DW)	(mg GAE g^−1^ DW)
2	30	20	8.10 ± 1.01 ^bcd^	2.43 ± 0.19 ^cde^
2	30	40	5.24 ± 0.34 ^e^	2.34 ± 0.24 ^e^
2	30	60	6.90 ± 1.35 ^de^	2.78 ± 0.07 ^bcde^
2	45	20	7.62 ± 0.00 ^cde^	3.02 ± 0.42 ^bcde^
2	45	40	7.50 ± 1.52 ^cde^	3.49 ± 1.02 ^bcd^
2	45	60	7.26 ± 0.17 ^cde^	3.16 ± 0.07 ^bcde^
2	60	20	7.98 ± 0.17 ^bcd^	3.95 ± 0.21 ^bc^
2	60	40	8.21 ± 0.17 ^bcd^	3.79 ± 0.06 ^bcd^
2	60	60	7.02 ± 0.17 ^de^	3.39 ± 0.37 ^bcde^
10	30	20	7.62 ± 0.00 ^cde^	1.90 ± 0.54 ^e^
10	30	40	6.90 ± 0.00 ^de^	2.38 ± 0.08 ^de^
10	30	60	8.21 ± 0.84 ^bcd^	2.55 ± 0.24 ^cde^
10	45	20	6.90 ± 1.01 ^de^	2.95 ± 0.56 ^bcde^
10	45	40	8.45 ± 0.17 ^bcd^	3.52 ± 0.03 ^bcd^
10	45	60	8.33 ± 0.67 ^bcd^	3.14 ± 0.18 ^bcde^
10	60	20	10.36 ± 0.51 ^ab^	3.22 ± 0.79 ^bcde^
10	60	40	9.76 ± 0.34 ^abc^	4.12 ± 0.07 ^b^
10	60	60	10.48 ± 0.34 ^ab^	3.77 ± 0.03 ^bcd^
2	95	-	7.62 ± 0.34 ^cde^	3.58 ± 0.21 ^bcd^
10	95	-	8.57 ± 0.34 ^bcd^	4.18 ± 0.19 ^b^
360	95	-	11.90 ± 0.34 ^a^	6.38 ± 0.28 ^a^

Means followed by different letters indicate a significant difference (*p* < 0.05).

**Table 3:** Project indices of the extraction processes.

*A. floribunda* Extract Selling Price (US$ flask^−1^)	Gross Margin (%)	Return on Investment (%)	Payback Time (years)	Internal Rate of Return after Taxes (%)	Net Present Value at 7.00% (US$ × 10^6^)
UAE					
4	3.43	9.85	10.15	-	−0.49
5	22.74	17.73	5.64	20.08	0.80
6	35.62	25.61	3.90	35.08	1.83
7	44.82	33.49	2.99	47.58	2.81
8	51.71	41.37	2.42	59.30	3.80
9	57.08	49.26	2.03	70.23	4.49
10	61.37	57.14	1.75	80.55	5.77
**HWE**					
4	1.95	3.38	10.66	-	−0.58
5	21.56	17.23	5.8	19.14	0.74
6	34.64	25.08	3.99	34.14	1.76
7	43.97	32.93	3.04	46.8	2.75
8	50.98	40.78	2.45	58.36	3.73
9	56.42	48.63	2.06	69.3	4.71
10	60.78	56.48	1.77	79.77	5.69
**SOXHLET**					
4	−45.77	−3.13	-	-	−2.10
5	−16.62	3.43	29.18	-	−1.29
6	2.82	9.54	10.48	-	−5.62
7	16.7	13.48	7.42	10.55	0.19
8	27.11	17.41	5.74	19.45	0.74
9	35.21	21.35	4.68	27.42	1.26
10	41.69	25.28	3.96	34.61	1.76

## Data Availability

Data included in article have been published on https://data.mendeley.com/datasets/stk2zv5dwd/1 (accessed on 6 September 2022).
